# When Viruses Cross Developmental Pathways

**DOI:** 10.3389/fcell.2021.691644

**Published:** 2021-08-05

**Authors:** Pankaj Trivedi, Sandesh Kumar Patel, Diana Bellavia, Elena Messina, Rocco Palermo, Simona Ceccarelli, Cinzia Marchese, Eleni Anastasiadou, Lisa M. Minter, Maria Pia Felli

**Affiliations:** ^1^Department of Experimental Medicine, Sapienza University of Rome, Rome, Italy; ^2^Department of Molecular Medicine, Sapienza University of Rome, Rome, Italy; ^3^Department of Veterinary and Animal Sciences, University of Massachusetts Amherst, Amherst, MA, United States

**Keywords:** Hedgehog, Notch, WNT, oncogenic viruses, immune evasion, microRNA, targeted therapies

## Abstract

Aberrant regulation of developmental pathways plays a key role in tumorigenesis. Tumor cells differ from normal cells in their sustained proliferation, replicative immortality, resistance to cell death and growth inhibition, angiogenesis, and metastatic behavior. Often they acquire these features as a consequence of dysregulated Hedgehog, Notch, or WNT signaling pathways. Human tumor viruses affect the cancer cell hallmarks by encoding oncogenic proteins, and/or by modifying the microenvironment, as well as by conveying genomic instability to accelerate cancer development. In addition, viral immune evasion mechanisms may compromise developmental pathways to accelerate tumor growth. Viruses achieve this by influencing both coding and non-coding gene regulatory pathways. Elucidating how oncogenic viruses intersect with and modulate developmental pathways is crucial to understanding viral tumorigenesis. Many currently available antiviral therapies target viral lytic cycle replication but with low efficacy and severe side effects. A greater understanding of the cross-signaling between oncogenic viruses and developmental pathways will improve the efficacy of next-generation inhibitors and pave the way to more targeted antiviral therapies.

## Introduction

About 15% of all human tumors have infectious etiology and yet only a handful of viruses are known to promote tumor development (White et al., 2014). Clearly, tumor development is neither the aim of the virus nor it is required for virus transmission. The evolutionary mechanisms of viral replication, establishment of latency and immune evasion are often the underlying causes that make the infected cell emancipate from normal proliferation dictated by homeostasis. Discovering oncogenic virus-targeted genes and developmental signaling pathways is imperative to understanding viral tumorigenesis. Viruses hijack different cellular programs with the aim to survive and replicate within its host. By interacting with host proteins, they perturb and interfere with host signaling pathways to modify critical cellular functions. Integration of the viral genome into the host DNA may be a critical factor in carcinogenesis, particularly for some Human Papilloma virus (HPV) serotypes (Oyervides-Muñoz et al., 2018). Herpesviruses on the other hand, mainly remain as extra-chromosomal DNA in the host cell nucleus. In both cases, viruses alter expression and function of genes primarily associated with cell proliferation, differentiation, and survival, and induce chromosomal instability chromosomal instability.

In recent years, computational reconstruction of proteome-wide protein–protein interaction (PPI) networks between viruses and developmental pathways have enhanced our understanding of virus-induced carcinogenesis. Mei and Zhang studied PPI networks to highlight important relationships between Epstein-Barr virus (EBV) proteins and developmental pathways, including Hedgehog (HH) and Notch signaling (Mei and Zhang, 2016). By exploiting components of the HH pathway, viruses promote tumor growth, survival, and stemness-associated programs in order to transform infected cells. Therefore, HH-targeted therapies could represent a promising strategy to combat virus-induced tumors. Another study demonstrated that Hepatitis B Virus (HBV) DNA integration preferentially targets cancer related pathways such as MAPK, extracellular matrix (ECM)-receptor interactions, and the HH signaling pathways (Yang et al., 2018).

The Notch pathway is evolutionarily conserved and participates in a plethora of physiological intercellular and intracellular signaling processes during differentiation and development of an organism. Evidence shows that virally perturbed Notch signaling may lead to cancer (Meisel et al., 2020). Oncogenic viruses also exploit Notch pathway to escape immune recognition and facilitate their own survival in the host to enhance infectivity and transmission. Remarkably, several viral oncoproteins, such as Epstein-Barr nuclear antigen 2 (EBNA2), Hepatitis Bx (HBx), latency-associated nuclear antigen (LANA) of Kaposi’s sarcoma-associated herpes virus (KSHV, also known as HHV-8) and others, interact with several members of the Notch pathway (Hayward, 2004).

The WNT signaling developmental program is also frequently targeted by oncogenic viruses to transform the target cells. HBV, Hepatitis C virus (HCV), EBV and Human T Lymphocyte Virus-1 (HTLV-1) co-opt or modulate components of the WNT pathway to effectively subvert normal cellular processes including cell proliferation, differentiation, and survival.

Thus, as our understanding of the mechanisms that regulate oncogenic transformation grows, the extent and complexity of cellular processes targeted by oncoviruses is better appreciated. The recognition that multiple developmental pathways are frequently targeted, either individually or collectively, may represent unexplored opportunities for developing unique or synergistic therapeutic strategies to treat or prevent viral tumorigenesis.

In this review, we present an overview of the three developmental pathways, namely, HH, Notch, and WNT and how some oncogenic viruses interact with them. We will review immune system interactions with these viruses, and how they regulate these pathways through viral miRNAs to survive and contribute to carcinogenesis and tumor progression. We will also provide perspectives for the development of therapies that target important and common regulators of these three developmental pathways.

## HH Signaling in Viral Oncogenesis

### Overview of HH Signaling

**FIGURE 1:**
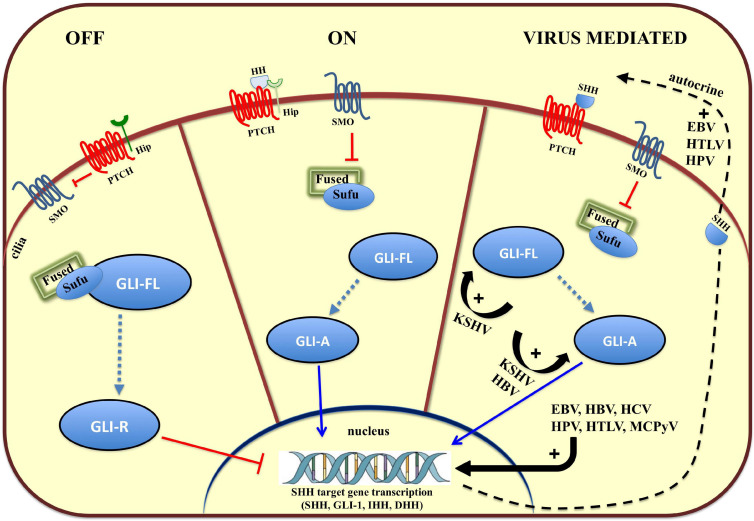
Overview of the Hedgehog (HH) signaling pathway and modulation by oncoviruses. On the left, in the absence of HH ligands (SHH, IHH, and DHH), the HH receptor Patched (PTCH) keeps the pathway off (OFF) by inhibiting Smoothened (SMO), and keeping the glioma-associated oncogene (GLI) transcription factors in an inactive form (GLI-R). In the middle, binding of HH ligand to HH receptor, PTCH, turns the pathway on (ON), and inhibits its activity, relieving the repression of SMO, which converts full-length GLI (GLI-FL) into a transcriptional activator (GLI-A). In vertebrates, cilia are required for production of GLI-repressor (GLI-R) and/or GLI-activator (GLI-A). On the left, viruses hyperactivate HH signaling with multiple mechanisms (VIRUS MEDIATED). EBV, Epstein Barr Virus; HBV, Hepatitis B Virus; HCV, Hepatitis C Virus; HPV, Human Papilloma Virus, KSHV, Kaposi’s Sarcoma-associated Herpes Virus; MCPyV, Merkel Cell Polyoma Virus.

Tissue patterning, cell differentiation and proliferation require HH signaling but aberrant HH signaling is an important cause of cancer. In humans, the HH pathway is activated by three ligands: Sonic Hedgehog (SHH), Indian Hedgehog (IHH), and Desert Hedgehog (DHH) (Qi and Li, 2020). These ligands share a high degree of sequence and functional homology and act to initiate this paracrine signaling cascade. The two Patched genes, PTCH1 and PTCH2 in humans, code for the primary receptors of HH ligands, consisting of 12 transmembrane helices (TMs), three extracellular domains (ECDs), and one C-terminal domain (CTD). The two receptors share a conserved TM domain and two of three ECDs. In contrast, PTCH2 lacks the CTD domain. After binding HH, PTCH1 is inhibited and forms oligomers, which are further moved out of the cilia and degraded in the endosome (Tukachinsky et al., 2016). In so doing, Smoothened (SMO) is no longer inhibited and this Frizzle-class G protein-coupled receptor (GPCR) can relocate to the cilia, a small organelle extending from the plasma membrane, which provides a localized hub in which transmembrane receptors can concentrate (Figure 1). SMO signals through at least two effector routes. The first is a G protein-independent, canonical pathway that signals to three members of the glioma-associated (GLI) oncogene family, with the aim to upregulate target genes (Figure 1). One of them, Hip (Figure 1), a HH interacting protein attenuates ligand diffusion (Arensdorf et al., 2016). In vertebrates, suppressor of Fused (SUFU) represses GLI transcription factor activation and the active SMO releases this inhibition. GLI1 functions as a feed-forward activator to sustain or amplify target gene expression (Pandit and Ogden, 2017). GLI2 and GLI3 are bifunctional and can be processed to act either as transcriptional activators or repressors (Crompton et al., 2007). Ciliary SMO signaling halts GLI processing, further stabilizing GLI2 and GLI3 as transcriptional activators of SHH target genes, such as GLI1. The second route, referred to as the non-canonical SMO signaling pathway, triggers transcription-independent responses that are fundamental to establishing and maintaining distinct cell behaviors during development (Pandit and Ogden, 2017). Involvement of the non-canonical SMO signaling in viral carcinogenesis is yet to be fully explored (Palle et al., 2015).

### HH Signaling Targeted by Oncogenic Viruses Promotes Tumor Development

In addition to its indispensable role in developmental processes, more than 25% of all cancers require autocrine or paracrine HH signaling as a fundamental supporter of tumor cell growth and survival (Figure 1) (Lum and Beachy, 2004; Iriana et al., 2021). In EBV-positive nasopharyngeal carcinomas (NPC) and EBV-infected epithelial cell lines, the virus can activate the HH signaling pathway through autocrine induction of SHH (Port et al., 2013). This is corroborated by the expression of HH pathway effectors (GLI1 and GLI2) and HH target genes, such as *PTCH1*, *FOXM1*, and *WNT5A*, a highly evolutionarily conserved non-canonical WNT ligand. Altered HH signaling is common in NPC and specifically a reduced expression of SUFU has been detected in a large number of NPC specimens (Port et al., 2013). An HH autocrine signaling loop has also been associated with HPV infection, a ‘primary hit’ in cervical cancer (CC) development. Tumor cells express HH pathway components, and HH signaling promotes proliferation, survival, and migration of CC cells. These pro-survival and protective roles are prevented by a small molecule inhibitor that blocks binding of Gli to DNA, GANT-61, which induces caspase 3 cleavage indicating an increased apoptosis in human CC cell lines (Samarzija and Beard, 2012).

Viruses can also epigenetically affect factors involved in cellular HH activation, which implies that they may participate directly in configuring chromatin architecture. This is the case of a malignant T cell disorder caused by infection with the human retrovirus, HTLV-1. Indeed, in adult T cell leukemia (ATL), the HTLV-1 TAX transcription factor epigenetically upregulates Ellis Van Creveld (EVC) family members, EVC1 and EVC2, both of which have been associated with the cellular HH activity and thus provides the pro-survival attributes of ATL cells (Takahashi et al., 2014). Additionally, TAX can also induce SHH transcription in an NF-κB-dependent manner to sustain HH autocrine stimulation in malignant cells (Figure 1). Besides directly activating HH signaling, oncogenic viruses may exploit the connection between the transcription factor, ZIC2, and the microRNA (miRNA), miR-129-5p, as recently reported in lymphangiogenesis and lymph node metastasis during NPC progression (Yu et al., 2020). Importantly, it has been demonstrated that EBV, a virus closely associated with NPC, may down-regulate miR-129 expression (Forte and Luftig, 2011). This leads to ZIC2 activation, a zinc-finger transcription factor that upregulates HH related signaling molecules, SMO, GLI1, and SHH. Additionally, Yu et al. (2020) demonstrate that miR-129-5p overexpression silences ZIC2 and decreases NPC cell proliferation, migration, and invasiveness, suggesting that miR-129-5p may serve as a novel therapeutic tool for NPC.

Infection with oncogenic viruses may be silent for years and HH pathway reactivation later in life has been associated with tumor development (Kuromi et al., 2017). Merkel cell polyomavirus (MCPyV) is detected in approximately 80% of Merkel cell carcinoma (MCC) (Harms et al., 2018). It is an aggressive neuroendocrine skin cancer that mainly affects the elderly. In this case, higher expression of SHH and GLI1 were significantly associated with a favorable prognosis and represent useful markers of MCC (Kuromi et al., 2017). Intriguingly, the interaction between MCPyV and developmental pathways has remained hitherto unexplored.

In EBV-associated tumors, the virus persists in one of the three distinct latent phases, each characterized by expression of a set of viral genes. In NPC, EBV latent gene expression is restricted to EBNA1, EBV-encoded RNA1/2 (EBER1/2), BARF1, the BamHIA transcripts (BARTs), as well as variable expression of genes encoding oncogenic membrane proteins LMP1 and LMP2. In these tumors, EBNA1, LMP1 and LMP2 all stimulate HH signaling, but only the latter two are capable of reprogramming cells toward stemness. This would suggest that each viral protein can trigger different molecular pathways involved either in tumor cell growth and survival, or in stemness-associated programs. Just like normal stem cells, cancer cells have the ability to self-renew, differentiate in different cell types and under specific tumor microenvironmental condition, dedifferentiate backward a primitive state. This feature provides tumor cells the ability to grow, metastasize and home to specific tissues. Given its ability to activate the WNT and Notch pathways, Port et al. (2013) suggested LMP2A may impose stem-like characteristics on EBV-infected epithelial cells by recruiting additional onco-developmental pathways. Indeed, the HH pathway, together with WNT and Notch signaling, maintain a population of normal stem cells and transforms cancer stem cells by inducing stemness-associated gene expression (Katoh, 2007). Overall, EBV and other oncogenic viruses trigger the HH autocrine signaling loop, a fundamental mechanism in cell survival and proliferation.

### HH Pathway Mediates Virus-Induced Epithelial to Mesenchymal Cell Transition (EMT)

Co-expression of cancer stem/initiating and mesenchymal cell markers have been observed in peritumoral stromal tissues within nodules of Hepatocellular Carcinoma (HCC) from livers of patients with chronic hepatitis. Both HBV and HCV clearance rates are high in infected individuals, often depending on the age (Chou et al., 2015). Only about 5–10% infected individuals become chronic carriers. In case of HBV, integration of the viral genome plays an important role in both chronic carrier state and subsequent diseases (Chou et al., 2015). Indeed, the chronically infected individuals have higher risk of developing HCCs. Other genetic events like mutation and/or inactivation of p53 are also frequent (Feitelson et al., 1993). HBx protein of HBV directly binds p53 and sequesters it in the cytoplasm thus compromising its ability to induce apoptosis (Elmore et al., 1997).

The HH signaling pathway is maintained in an inactive state in a healthy adult liver due to high levels of Hip, which disrupts engagement between the HH ligand and the receptor (Hyun and Jung, 2016). Gene profiling studies of human liver cancers provide evidence that chronic infection by HBV and HCV significantly increases hepatic mRNA expression of HH-ligands, *SHH* and *IHH*, and target genes, *PTCH* and *GLI2*, possibly during more advanced stages of liver disease (Pereira Tde et al., 2010). Indeed, Pereira Tde et al. (2010) reported that the fibrosis stage and HCC development are predicted to increase in parallel with the level of HH pathway activity. As the level of HH ligands increases, hepatic accumulation of HH-reactive progenitors also increases, concurrent with a decreased Hip expression (Hyun and Jung, 2016). The EMT transition is characterized by epithelial cells losing their polarity, the ability of cell-cell contact and acquiring features which make them resemble mesenchymal cells. While EMT occurs physiologically during embryogenesis, this transition is also a characteristic of many neoplastic diseases. The loss of E-cadherin by epithelial cells is considered the cornerstone of EMT (Kalluri and Weinberg, 2009). As such, Pereira Tde et al. (2010) reported that an increased activity of HH signaling in chronic viral hepatitis correlates to the enriched sub-population of HH-responsive progenitors that are undergoing EMT in hepatic nodules. Cellular migration is an integral step in EMT that results in liver remodeling in chronic liver disease and promotes metastasis during cancer progression (Arzumanyan et al., 2012). This event is largely dependent on activation of HH signaling (SHH and GLI2) by the HBV transcription factor, HBx, in HCC pathogenesis. The mechanism by which HBx upregulates the expression of HH components, either through transcriptional control (Arzumanyan et al., 2012) or through post-translational stabilization and nuclear localization (Kim et al., 2011), remains to be fully elucidated. Moreover, HBx also promotes stemness in the liver (Arzumanyan et al., 2011). Perhaps, the discovery that HBx activates HH signaling in the pathogenesis of HCC may lead to therapies that are better targeted to prevent tumor initiation and/or that block the growth and relapse of established tumors. Interestingly, in hepatic carcinogenesis EMT and enhanced HH signaling activation have been suggested to promote chemoresistance and invasion of poorly differentiated hepatoma cells often negative for CD133 and EpCAM. These observations may provide a new basis for reclassifying HCC specimens and may represent promising targets in eradicating chemoresistant subpopulations in HCC (Chen et al., 2011).

### Developmental Pathways Are Not the Only Downstream Targets of Oncogenic Viruses

Although the role of viral oncoproteins E6 and E7 in HPV-mediated cervical carcinogenesis is well-established, still to be studied is the interaction of GLI signaling with HPV encoded oncogenes. Recently, active GLI signaling has been demonstrated in CC cells irrespective of the presence of HPV and was associated with cell viability. Inhibiting GLI signaling in HPV-positive CC cells is associated with reduced HPV E6 oncogene expression and loss of stemness (Vishnoi et al., 2016). Conversely, silencing the HPV-16 E6 oncogene reduced *GLI1* transcription. This reciprocal interference suggests a cooperation between viral and HH proteins. Indeed, inhibiting both E6 and GLI signaling produces an additive effect on cell viability, leading to the hypothesis that they synergize to promote stemness in CC cells. Loss of p53, triggered by HPV E6, is a probable connecting link with constitutively active GLI signaling observed during persistent high risk HPV infection. Additionally, GLI-HPV E6 cooperation sustains cancer cell stemness possibly leading to tumor progression and chemoresistance, as observed in clinically advanced CC (Vishnoi et al., 2016).

According to Piirsoo et al. (2019) full-length GLI has a role in regulating HPV transcription and, surprisingly, it acts as a repressor. Human GLI1 has at least three alternatively spliced isoforms, including GLI1ΔN that lacks the conserved phosphorylation cluster and the SUFU binding motif. Shuttling of GLI1 between the cytoplasmic and nuclear compartments depends on several factors, including interaction with SUFU and Protein Kinase A-mediated phosphorylation. These authors further demonstrate that, in contrast to GLI1ΔN, full-length GLI1 suppresses replication of multiple HPV genomes, including HPV-5, -11, and -18. Non-productive infections often terminate the viral life cycle and may be crucial for HPV-DNA persistence and tumorigenesis (Gaglia and Munger, 2018). According to the bioinformatic analysis, the predicted GLI1 binding site mostly overlaps with the viral E2 binding site, suggesting a direct impact on the initiation of HPV5 replication. Overall, this observation suggests there may be differing contributions of GLI1 isoforms to the HPV lifecycle and, interestingly, highlights the regulatory role of Gli1 on HPV transcription.

Viral carcinogenesis may also rely on the ability of oncogenic viruses to select a suitable genetic cell environment without directly targeting HH signaling. Cervical carcinogenesis is a multistep process. HPV infection is not sufficient *per se*, but provides a ‘second hit,’ most likely through moderate levels of Notch1 and the cooperation of HH and WNT signals to transform keratinocytes (Lathion et al., 2003). In this context, HH signaling is not triggered directly by HPV E6 and E7 proteins but, rather, that HH-activating mutations are selected in cells initially immortalized by HPV (Samarzija and Beard, 2012). Therefore, the dual role of HH signaling, serving either as a collaborator of HPV-induced carcinogenesis or as a regulator of viral oncogene expression stresses the critical role of HH inhibitors as a therapeutic option in CC.

### Virus-Induced Tumor Progression and Immune Evasion Strategies Point to GLI1 Activity

Recently, Asha et al. (2020) reported on the unexplored function of HH signaling in regulating the biology of latent and lytic states of sarcoma KSHV. This virus, also known as human herpesvirus 8 (HHV8) hijacks pro-inflammatory pathways and concurrently reduces anti-inflammatory Lipoxin A4 (LXA4) secretion to maintain the virus in a latent state. In KS skin tissue, GLI1 is significantly increased and is distributed both in the cytoplasm and nucleus (Figure 1). This is in contrast to healthy tissue in which GLI1 is expressed exclusively in the nuclei. LXA4-treated KSHV-infected cells showed decreased GLI1 expression, independent of SHH modulation, and mainly through GLI1 destabilization, which may also decrease the angiogenic processes. In fact, GLI1 can transcriptionally upregulate vascular endothelial growth factor C (VEGF-C) expression to promote angiogenesis (Carpenter and Lo, 2012). To this end, the GLI1 antagonist, GANT-61, can reduce tumor formation by a KS-derived cell line. The sphere-forming efficiency, as well as the average volume of the formed spheres, were significantly decreased in GANT-61-treated cells, suggesting GLI1 inhibitors may act to attenuate tumor formation during KS initiation or progression. Immune evasion strategies, such as HLA class I downregulation is frequent during the progression of human tumors. In EBV-associated gastric cancer, latency I viral genes are often expressed (Deb Pal and Banerjee, 2015). Among the latency I viral genes, *EBERs* and *LMP2A* are frequently detected in most gastric cancer samples, suggesting the virus expresses only cell context-adapted genes. It has been reported that LMP2A exploits the HH pathway by activating SHH signaling to induce HLA class Ia downregulation in gastric cancer cells (Deb Pal and Banerjee, 2015). LMP2A-induced downmodulation or complete loss of HLA class Ia expression was specifically mediated by elevated GLI1 protein expression. Furthermore, inhibition of other important self-renewal pathways such as Notch, WNT, or PI3K, in LMP2A-expressing gastric cancer cells could not prevent HLA class I down-regulation, providing evidence in support of the hypothesis of cell-context and signaling-specific requirements by the virus (Deb Pal and Banerjee, 2015).

Overall, viruses can impinge on HH signaling in several ways. For example, EBV, HPV, HTLV, MCPyV, HBV, and HCV can trigger an autocrine SHH signaling loop to promote tumor development and stemness-associated programs. Instead, KSHV triggers Gli-mediated angiogenesis (VEGF) without involving SHH. The hepatotropic HCV and HBV can cause chronic inflammation and turn on an inactive HH signaling in the healthy liver to promote fibrosis and HCC development. Another mode of action to perturb HH signaling is through alteration of Gli transcription as highlighted by HPV encoded E6 protein.

Autocrine stimulation plays an indispensable role in HH-mediated viral carcinogenesis, but its effect in the tumor microenvironment is still uncovered. Future studies are required to investigate how viruses utilize and relocate HH family members in infected cells to drive host cell machinery to carcinogenesis.

## Notch Signaling in Viral Oncogenesis

### Overview of the Notch Pathway

Morgan (1917) observed a “notch” in the wings of a mutant *Drosophila*. It was subsequently found to be linked to a heterozygous deletion of a gene located on the chromosome X, hence named *Notch.* The Notch signaling cascade is highly conserved from *Drosophila* to humans (Artavanis-Tsakonas et al., 1999), and consists of receptors, ligands, and intracellular proteins that transmit the signals to the nucleus. The four mammalian Notch receptors (Notch1-4) are large Type I transmembrane proteins. A furin-like convertase catalyzes the proteolytic maturation of Notch receptor pro-proteins in the Golgi apparatus (Logeat et al., 1998). The proteolysis generates two subunits connected by Ca^2+^-dependent ionic bonds: the Notch extracellular domain (NECD), consisting of multiple epidermal growth factor-like (EGF) repeats which mediate ligand binding, and the Notch intracellular domain (NICD), which is the transcriptionally active part of the molecule (Gordon et al., 2008; Yavropoulou and Yovos, 2014). NICD translocates to the nucleus where it binds Recombination signal binding protein for immunoglobulin kappa J region (RBP−Jκ) and, in cooperation with Mastermind-like 1 (MAML1), regulates the transcription of Notch target genes (Borggrefe and Oswald, 2009; Borggrefe et al., 2016; Roo and Staal, 2020). Interestingly, MAML1 is a versatile coactivator in other signaling pathways too. Indeed, it can serve as a Notch-independent transcriptional activator in the HH, Wnt/β-catenin and Hippo signaling pathways (Quaranta et al., 2017; Zema et al., 2020). The Notch pathway signaling can be “canonical” or “non-canonical,” based on whether NICD interacts with RBP−Jκ or not (Ayaz and Osborne, 2014). Canonical Notch ligands are also Type I transmembrane proteins and belong to the Delta/Serrate/LAG-2 (DSL) family of proteins. Considered to be the structural homologs to the Delta and Serrate ligands of *Drosophila*, mammalian Notch ligands are Delta-like proteins, named Dll1, Dll3, and Dll4, and Serrate homologs known as Jagged1 and Jagged2 (JAG1 and JAG2) (D’Souza et al., 2008; Kopan and Ilagan, 2009). Independently of Notch, Jagged1 can induce intrinsic reverse signaling within the ligand-expressing cell as demonstrated in CC cells (Pelullo et al., 2019). One non-canonical Notch signaling example is the regulation of Wnt/β-catenin signaling, which uses β-catenin as a transcriptional mediator (Sanders et al., 2009; Andersen et al., 2012).

Ligand-receptor interactions and transcription factor activity in Notch signaling play pivotal roles in a wide variety of differentiation processes, including regulation of cell-fate determination during tissue and cell development (Wang et al., 2015; Chandiran et al., 2018; Ferrandino et al., 2018b). Notch signaling affects proliferation, apoptosis, and cell differentiation (Grazioli et al., 2017). Moreover, active Notch signaling allows cells to maintain stem-cell-like features (Kopan, 2012; Kessler et al., 2015; Moriyama et al., 2018).

### Notch Signaling in Viral Oncogenesis

**FIGURE 2:**
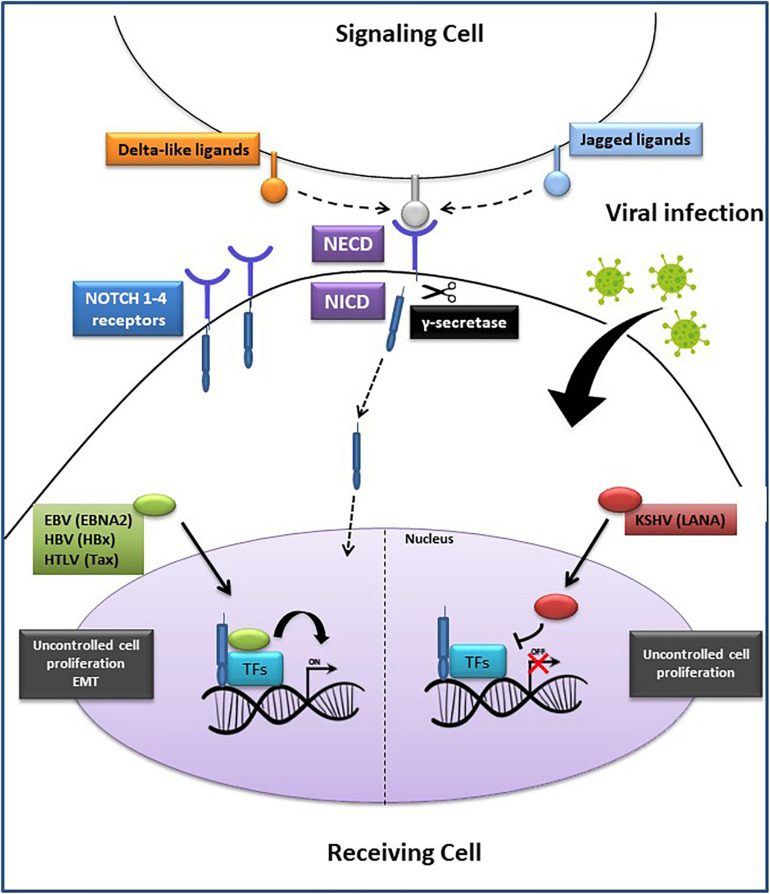
Oncogenic viruses exploit the Notch signaling pathway. Notch ligands (Delta-like or Jagged) on the signaling cell bind to Notch 1–4 receptors on the receiving cell, generating the Notch extracellular domain (NECD) and the Notch intracellular domain (NICD). Subsequently, the proteolytic cleavage of NECD by γ-secretase, generates NICD translocation into the nucleus where it binds with the transcription factors (TFs): recombining binding protein Jk (RBP–Jκ), cAMP response element-binding protein (CREB), forming an active complex that regulates the transcription of Notch target genes. On the **(Left side)**: EBNA2, HBx and Tax proteins encoded by EBV, HBV, and HTLV, respectively, promote cell proliferation or epithelial-mesenchymal transition (EMT) through a direct interaction with the transcription complex, leading to tumorigenesis. On the **(Right side)**: LANA protein, encoded by KSHV, prevents the activation of the transcription complex, resulting in an accumulation of NICD and increased cancer cells proliferation. EBV, Epstein Barr Virus; HBV, Hepatitis B Virus; HTLV-1, Human T Lymphocyte Virus-1; KSHV, Kaposi’s Sarcoma-associated Herpesvirus.

Perturbing the Notch pathway can lead to the onset of various diseases, including cancer (Sjölund et al., 2005; Penton et al., 2012; Takebe et al., 2015; Krump and You, 2018). Co-opting and dysregulating developmental pathways by oncogenic viruses also involve targeting Notch signaling components (Figure 2) (Krump and You, 2018). HBV proteins activate Notch signaling to stimulate uncontrolled cell proliferation, which then may lead to HCC (Mesri et al., 2014). The HBV encoded protein, HBx, is one of the key viral factors capable of malignantly transforming infected cells. It upregulates Notch1 receptor, either through a direct interaction or through the p38 MAPK pathway, to promote HCC proliferation (Kong et al., 2016). This viral protein also stimulates Notch1-4 expression. The cytoplasmic Notch1 and the nuclear Notch4 correlate with HBx expression in HCC tissues (Gao et al., 2016). HBx-Dll4-Notch1 axis seems to have a critical role in regulating cell survival in HCC. Indeed, HBx mediates Dll4 upregulation, which increases Notch1 cleavage, thus activating Notch signaling (Kongkavitoon et al., 2016). Crosstalk between HBx and JAG1 in HCC has also been documented. JAG1 is highly expressed in HCC tissues and is regulated by HBx, further confirming an oncogenic role of the latter in activating Notch signaling (Gao et al., 2007). HBx involvement in HCC pathogenesis was further demonstrated by the discovery of a regulatory axis between this viral protein and miR-3188. This oncogenic miRNA is overexpressed in HCC tissue and knocking-out miR-3188 using CRISPR/Cas9 de-repressed expression of its target (zinc fingers and homeoboxes 2) (ZHX2), a transcriptional repressor of Notch1. Thus, at least one way by which miR-3188 acts to induce Notch signaling and promote HCC pathogenesis is by negatively regulating ZHX2 (Zhou et al., 2017).

The EBV encoded nuclear protein EBNA2 is a biological equivalent of Notch1 (Zimber-Strobl and Strobl, 2001). Interestingly, this viral protein interacts with the same cellular repressor RBP-Jk as does NICD. EBNA2 and NICD, both have activating domains that interact with RBP-Jk transcriptional repressor, causing HDAC replacement leading to viral and cellular gene transcription (Strobl et al., 1997). Histone acetyltransferases (HATs), such as P300/CBP-associated factor (PCAF), also interact with EBNA2 and NICD in the context of gene transactivation. Constitutive activation of Notch1-4 in different cell types can lead to tumorigenesis (Aster et al., 2017; Tottone et al., 2019). Since Notch1 signaling is associated with cancer, it is significant that EBNA2 can hijack components of this pathway to immortalize and transform B cells into lymphoblastoid cell lines (LCLs) (Tierney et al., 2015). In a reciprocal experiment, Strobl et al. (2000) have shown that activated Notch1 (N1ICD) can substitute for some EBNA2 functions. Specifically, they observed that in stably transfected Burkitt lymphoma (BL) cell lines carrying EBNA2-deletion, N1ICD was able to induce expression of some but not all EBNA2-inducible genes, such as *c-myc*, *CD21*, and *LMP2A*, but not *LMP1* or *CD23* (Strobl et al., 2000). These observations have led to the hypothesis that in those EBV associated cancers where EBNA2 is not expressed, NICD signaling can play a very critical role in transformation (Höfelmayr et al., 2001; Zimber-Strobl and Strobl, 2001; Chiara et al., 2016). A previous study showed that EBV-immortalized LCLs are characterized by high levels of telomere-specific reverse transcriptase (TERT), a catalytic component of telomerase, which prevented the switch from latency to lytic cycle activation of EBV (Giunco et al., 2015). The underlying mechanism involved activation of Notch2, which in turn, induced the Basic Leucine Zipper ATF-like (BATF) transcription factor. BATF negatively regulated BZLF1, the master regulator of the EBV lytic cycle, thereby preserving the latent state of the virus and survival of the infected B cells (Giunco et al., 2015).

KSHV can also subvert Notch signaling to promote survival of KSHV-infected primary B cells (Lan et al., 2006). One of its latent proteins, LANA, interacts with the tumor suppressor Sel10-mediated ubiquitin-proteasome pathway, which negatively regulates NICD. A study showed that LANA sequesters Sel10 by forming a complex in primary effusion lymphoma (PEL) cells (Lan et al., 2007). This complex prevents Sel10-NICD interactions, resulting in stabilized NICD, increased cell proliferation and angiogenesis. Moreover, KSHV-encoded replication and transcription activator (RTA) induced JAG1 expression, thereby activating Notch signaling. This leads to inhibition of lytic reactivation in a pro-lytic tumor microenvironment, maintaining the balance between lytic and latent state in KSHV infected cells, and ultimately resulting in virus-immune escape and persistence in the host (Li et al., 2016).

A recent study showed that in HPV-related CC, the Notch pathway is indeed affected by aberrant mutations, amplifications, and deletions (Yang et al., 2020). A copy number variation analysis (CNV) was performed in 282 CC patients and among the affected genes, they found amplifications of 4q34.1 F-Box And WD Repeat Domain Containing 7 (*FBXW7*) and of 1p36.11 hairy and enhancer of split-1 (*HES*)*2/3/4/5* tumor suppressor genes belonging to the Notch pathway. Interestingly, this was associated with significantly improved overall survival (OS) of these patients. In spite of several important limitations, such as the lack of information about HPV-type associated with the aberrant amplification of the two tumor suppressor genes of the Notch pathway, this study provides new insights for the prognosis of CC patients, based on tumor cell methylation signatures.

Notch signaling is exploited by the HTLV-1 encoded Tax1. This viral transcription factor activates Notch1 and prolongs the half-life of NICD, in ATL cells (Cheng et al., 2019). Tax1 formed a ternary complex with NICD, and RBP-Jκ, thus promoting cell proliferation and tumor progression. Another study showed that HTLV-1-encoded Tax induced JAG1 in most ATL cell lines, through the transcription factor NF-kB (Bellon et al., 2018). The ATL cell lines which did not express high levels of JAG1 were investigated for the presence of post-transcriptional inhibitory mechanisms mediated by miRNAs. The lack of JAG1 protein expression in these cell lines was due to high expression of miR-124a, which directly binds to the 3′UTR of *JAG1* mRNA. Furthermore, in most ATL patients with high JAG1 expression, miR-124a levels were low. Interestingly, *STAT3*, and *NFATc1* were also highly expressed. Remarkably, they are both miR-124a targets. The absence of miR-124a in ATL might enhance JAG1 and Notch1 signaling pathway sustained by constitutive expression of *STAT3* and *NFATc1*. Therefore, inhibiting JAG1 could be a promising therapeutic strategy in ATL (Bellon et al., 2018).

Another virus that perturbs the Notch pathway is HCV. The virus encoded non-structural protein 3 (NS3) is essential for its replication and contributes to viral induced HCC (Iwai et al., 2011). The Yeast two-hybrid screening and co-immunoprecipitation assays in mammalian cells, showed that Snf2-related CBP activator protein (SRCAP) interacted with NS3 and both proteins activated the *Hes1* promoter, a downstream target of the Notch pathway. Thus, HCV NS3 together with SRCAP and a SRCAP-resembling protein, p400, activated the Notch signaling pathway. A more recent article has demonstrated report showed that Notch signaling and CD4 T helper 22 (Th22) cells are involved in chronic HCV pathogenesis (Jiang B.C. et al., 2017). Specifically, the Notch interaction with aryl hydrocarbon receptor (AhR) induced IL-22 production by Th22 cells, thus favoring persistent HCV infection. Another study reported that Notch1 and -2 enhanced regulatory T cells (Tregs) and Th17 cell functions thus facilitating HCV infection (Qin et al., 2017). These studies suggest that inhibiting γ-secretase and thus Notch activation, might enhance immune surveillance against chronic HCV infection by down-regulating the production of IL-22, IL-17, as well as Treg-mediated immune tolerance in patients with chronic HCV.

Since oncogenic viruses co-opt Notch pathway components to sustain their survival leading to tumor progression, the development of inhibitors to block virus-Notch interactions could become a valuable therapeutic approach. To this end, utilizing monoclonal antibodies, drugs targeting Notch receptors, γ-secretase inhibitors (GSI), or small molecules that disrupt the interaction between Notch and RBP-Jk, RBPJ INhibitor-1 (RIN1), provide novel therapeutic avenues to pursue. For instance, monoclonal antibodies (mAbs) specifically targeting Notch1 reduced stemness of breast cancer cells (Sharma et al., 2012), whilst blocking antibodies against JAG1 in colorectal cancer (CRC) patients provided therapeutic benefits with low intestinal toxicity (López-Arribillaga et al., 2018). Enoticumab, an anti-Dll4 mAb, administered to patients with advanced solid tumors, inhibited the growth of these tumors and in ovarian cancer (OC), in a dose-dependent manner (Chiorean et al., 2015). RIN1 inhibited RBP-Jk transcription and interaction with NICD, thereby reducing proliferation in T-ALL and mantle cell lymphoma (MCL) cell lines (Hurtado et al., 2019). Focusing on viral-Notch interactions, it has been shown that H1N1 influenza virus challenge in mice increased Notch ligand Dll1 expression on macrophages, dependent on retinoic acid-inducible gene-I (RIG-I), which in turn induced the type I IFN pathway. Inhibiting γ-secretase during viral infection resulted in decreased IFNγ production, increased H1N1 load and acute inflammation in mouse lungs (Ito et al., 2011). Treating primary and immortalized KSHV cells with the GSI, LY-411,575 induced apoptosis in these cells, revealing a therapeutic alternative for patients with KSHV related diseases (Curry et al., 2005). Additionally, given that Notch 2 plays an important role in maintaining EBV latency, the use of GSIs has been proposed as a therapeutic strategy for EBV associated lymphomas (Giunco et al., 2015).

With some exceptions mentioned above, such as in the case of H1N1, in which inhibiting the Notch pathway with GSI led to adverse effects in mice, the Notch inhibitors have the potential to be therapeutically implemented with necessary precautions to interrupt the Notch pathway activation by viral proteins.

### Viruses Exploit Notch Signaling to Escape Immune Responses

Immune escape by tumor cells is a fascinating phenomenon. Indeed, tumor development is, quintessentially, a failure of the immune system to recognize and eliminate the cells that have emancipated themselves from cell cycle control and have gone awry (Prendergast, 2008). The immune evasion is achieved in several different ways. For instance, downregulation of HLA molecules on the tumor cell surface, upregulation of inhibitory immune checkpoint proteins, alteration of tumor cell death pathways, or an increase in immunoregulatory and immunosuppressive cytokines within the tumor microenvironment (TME) are some examples how tumor cells become invisible to the immune control.

A paradigmatic example of how Notch pathway activation could be central to immune suppression is provided by EBV associated Hodgkin’s lymphoma (HL). This tumor is characterized by increased infiltration of regulatory T (Treg) cells. The characteristic Reed Sternberg (RS) cells, which represent the HL tumor component, highly express Notch1 and Notch2 (Jundt et al., 2002). Interestingly, the same cells also produce high levels of CCL22 (Ishida et al., 2006) and this chemokine is important for recruiting Tregs into the TME. It has also been shown that another chemokine, CCL20, is upregulated in HL by the EBV-encoded nuclear protein, EBNA1. Just as CCL22, CCL20 is also critical for the increased numbers of Tregs see in HL. Given that Notch signaling can significantly influence cytokine and chemokine profiles in tumor cells to alter the tumor landscape (Colombo et al., 2018), it will be important to investigate if increased Notch1 and 2 expression in RS cells might affect CCL22 and CCL20 expression to augment regulatory T cell recruitment in HL.

Non-coding RNAs, especially miRNAs, play a significant role in helping tumors escape immune control by negatively regulating critical immunomodulatory genes (Li et al., 2021; Mondal et al., 2021). MiR-346 targets transport associated protein 1 (TAP1) and reduces transport of peptides into the endoplasmic reticulum for binding to HLA class I molecules (Bartoszewski et al., 2011). MiRNAs are also involved in immune checkpoint regulation. Recently, we have shown that EBV-encoded EBNA2 can induce PD-L1 expression in lymphoma cells (Trivedi et al., 2018; Anastasiadou et al., 2019). The increase in PD-L1 was due to a decrease in miR-34a. Interestingly, miR-34a also targets Notch (Kang et al., 2013). Thus, it seems that EBNA2, in addition to acting as a functional homolog of NICD, may also keep Notch expression and activity high by downregulating miR-34a. It will also be important to examine whether Notch and PD-L1 expression are positively correlated.

**FIGURE 3:**
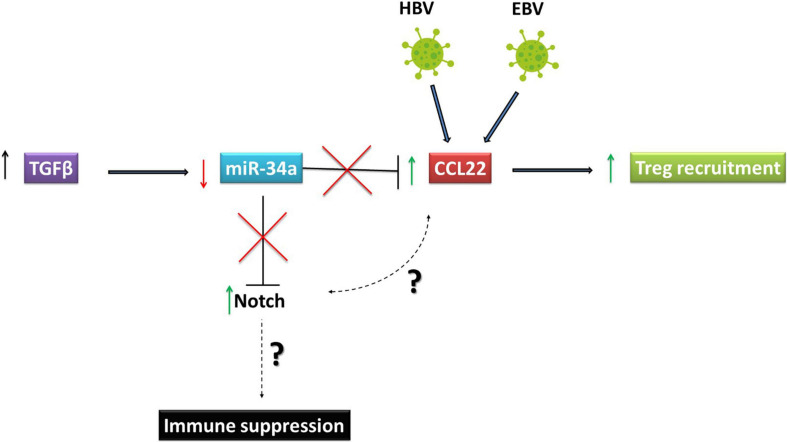
Oncoviruses exploit Notch signaling to escape immune responses. Reed Sternberg cells, which represent the Hodgkin’s Lymphoma tumor component are high expressers of Notch 1 and 2 and produce high levels of CCL22. This chemokine is important for recruiting regulatory T (Treg) cells to the tumor microenvironment. In HBV associated HCC, PVTT symptoms are positively correlated with TGFβ. The increase in TGFβ led to downregulation of miR-34a, which targets CCL22. Downregulation of miR-34a induced by TGFβ in HCC led to an increase in CCL22 and consequently in regulatory T cell recruitment to create an immunosuppressive tumor environment. HBV, Hepatitis B virusVirus; EBV, Epstein Barr Virus; HCC, Hepatocellular Carcinoma.

In HBV associated HCC, the metastatic event characterized by the portal vein tumor thrombus (PVTT), is common in over a third of patients. The positive correlation between the presence of HBV and PVTT has been established (Yang et al., 2012). These PVTT symptoms of HCC were also positively correlated with TGFb, where an increase in TGFb led to downregulation of miR-34a. Strikingly, CCL22 was shown to be an authentic target of miR-34a. Thus, TGFb-mediated miR-34a downregulation in HCC led to an increase in CCL22 and, consequently, to regulatory T cell recruitment to create an immunosuppressive tumor environment (Figure 3). The role of HBV in PVTT development is clear because over 82% of HBV positive HCC patients developed PVTT, compared with only 14% HBV negative HCC patients (Yang et al., 2012). As mentioned above, since miR-34a downregulates Notch, it will be critical to establish the link between high CCL22 and Notch expression and how Notch signaling may play an immunosuppressive role in HBV associated HCC. Furthermore, several studies have shown that aberrant activation of NICD in T cells may lead to T-cell acute lymphoblastic leukemia (T-ALL) confirming the importance of the Notch pathway in the progression of immune system-related malignancies (Ferrando, 2009; Bernasconi-Elias et al., 2016; Ferrandino et al., 2018a).

It is remarkable how diverse viral proteins, such as EBNA2, LANA and Tax1, are all able to deregulate the same effector of the Notch pathway, NICD, leading to cell transformation, latency maintenance, proliferation, angiogenesis and tumor progression. HBx and NS3 viral proteins interact with other activators of the Notch pathway, such as JAG1, Dll4, and SRCAP, respectively, to favor HCC progression. Continued investigation will enable a deeper understanding of how viral proteins interact with the Notch signaling to take advantage of host cells and escape immune surveillance. The knowledge of these interactions is an important step for the development of targeted therapies in virus-associated cancer patients.

## WNT Signaling in Viral Oncogenesis

### Overview of WNT Signaling

**FIGURE 4:**
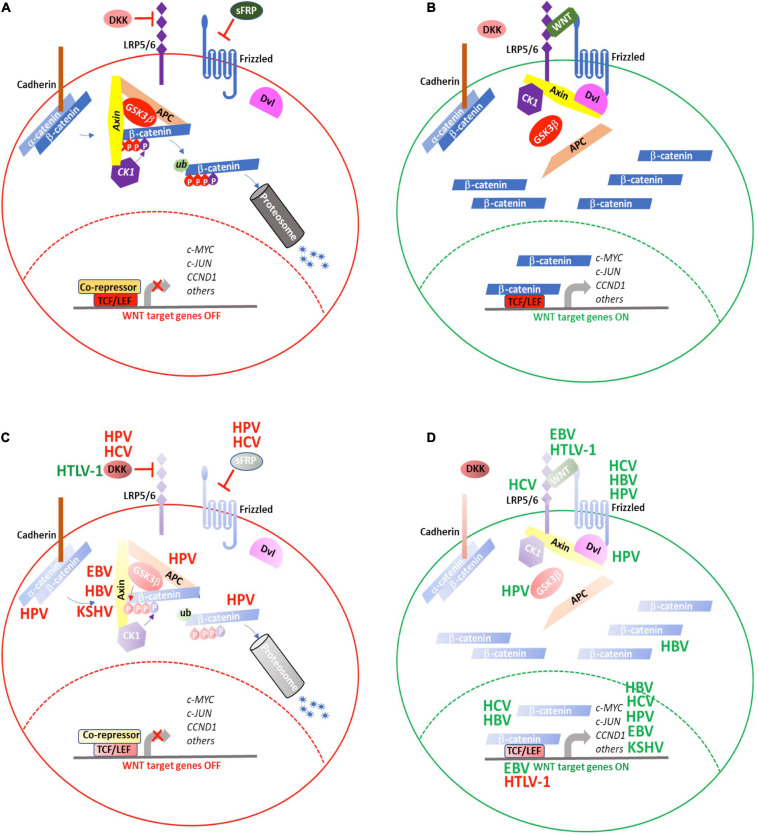
WNT signaling is targeted by oncogenic viruses during tumorigenesis. **(A)** WNT signaling is maintained in an “OFF” state by active processes that result in proteosomal degradation of cytosolic β-catenin and prevent transcription of WNT target genes. **(B)** Secretion of WNT ligands activate signaling though FZD and LRP5/6 to recruit the AxinAXIN-APC-GSK3αGSK3b complex away from β-catenin, resulting in its cytosolic stabilization and eventual translocation to the nucleus where it activates the TCF/LEF transcriptional complex to induce expression of WNT target genes. The oncogenic viruses, HBV, HCV, HPV, EBV, HTLV-1, and KSHV target various aspects of WNT signaling either to **(C)** repress inhibitors of or **(D)** enhance positive regulators of WNT signaling. (red) Negative regulation; (green) positive regulation. See text for details.

WNT signaling is involved in cell proliferation, cell polarity, and cell fate determination during embryonic development and tissue homeostasis. Evolutionarily, the WNT signaling pathway is highly conserved. Under normal or homeostatic conditions, in the absence of WNT ligands, the signaling is maintained in an “off” state (Figure 4A). Under this condition, b-catenin that is not membrane-associated is sequestered in a cytosolic complex together with Auxin, adenomatous polyposis coli (APC), casein kinase I (CKI), and glycogen synthase kinase (GSK) 3b. Within this complex, b-catenin is sequentially phosphorylated by CKI and GSK3b, which primes b-catenin for ubiquitination and proteasomal degradation (Stamos and Weis, 2013). This process of active degradation prevents b-catenin from translocating to the nucleus. In the absence of nuclear b-catenin, WNT target gene transcription is repressed when Transcription Factor (TCF) binds to WNT gene promoters in association with transcriptional co-repressors, such as TLE1 (Transducin-Like Enhancer of Split1).

WNT signaling is activated when secreted WNT proteins bind to the seven-pass transmembrane receptor, Frizzled (FZD), together with its co-receptor, low-density lipoprotein receptor related protein (LRP) 6, or to the closely related, LRP5. Following WNT binding, the FZD-LRP6 receptor complex associates with the Disheveled segment polarity protein (Dvl), a scaffold protein that facilitates LRP6 phosphorylation and subsequent recruitment of Axin way from APC and GSK3b to form the FZD-WNT-LRP6-Dvl-Axin multiprotein complex (Komiya and Habas, 2008). As the interactions of Axin with APC and GSK3b are diminished, the complex loses its ability to bind to b-catenin and mediate its degradation. As a result, b-catenin accumulates in the cytosol and, ultimately, translocates to the nucleus, where it successfully out-competes TLE for binding to TCF and activates WNT target gene transcription (Figure 4B).

WNT signaling regulation is complex. Many components of this pathway are, themselves, positively or negatively regulated through the WNT pathway. Through their co-evolution with the human immune system, oncogenic viruses have devised means either to down-regulate inhibitory WNT proteins, such as Dickkopf WNT signaling pathway inhibitor1 (DKK1) (Niida et al., 2004) (Figure 4C) or reinforce positive WNT signaling networks (Figure 4D) to promote cellular transformation and tumorigenesis.

### Oncogenic Hepatitis Viruses Modulate WNT Signaling

Oncogenic HBV and HCV infect cells of the liver causing hepatic inflammation, fibrosis, cirrhosis, and, ultimately, HCC. The progression from initial infection to HCC is a lengthy, complex, multi-step process. HBV and HCV are vastly different – HBV is a partially double-stranded DNA virus (Liang, 2009), while HCV is a positive-sense, single-strand RNA virus (Dubuisson and Cosset, 2014)– making it unsurprising that the molecular mechanisms by which they induce tumorigenesis also differ. However, evidence suggesting that both HBV and HCV hijack components of the WNT signaling pathway underscores the importance of subverting WNT signaling in HCC.

### Oncogenic Viruses Subvert WNT Signaling Through Multiple Mechanisms

Mutations in *CTNNB1*, the b-catenin encoding gene, is associated with HCC (Javanmard et al., 2020). However, genomic instability caused by HBV infection is more commonly associated with inactivating mutations in *AXIN1* leading to HCC (Li et al., 2013). The HBV genome consists of four overlapping genes encoding surface antigens (*S* gene), core proteins (*C* gene), HBV polymerase (*P* gene), and the HBx protein (*X* gene). The polymerase lacks proofreading activity, so HBV mutations occur with greater frequency than other DNA viruses. Frequently, these mutations are associated with activation of WNT signaling. Mutations in the core promoter, overlapping with the *X* gene, have also been described. The resulting single or combination point mutations in HBx, which is thought to act as a promiscuous transcription factor, also upregulated WNT signaling (Chen et al., 2016). Overexpressing HBx mutants in human hepatocyte cell lines increased phosphorylated GSK3b, in the absence of increased total GSK3b. This suggests HBx mutants may be acting upstream of GSK3b to regulate its phosphorylation and subsequent inactivation, although whether this occurs as a result of Srk or Erk kinase activation or through its interaction with *APC*, remains to be fully elucidated. Functionally, cells expressing HBx mutants showed greater proliferation and migration, which could be abrogated by co-expressing siRNA to b-catenin. HBx mutant cells showed increased expression of cytoplasmic and nuclear b-catenin and, importantly, HCC patient samples with combination mutations also showed high levels of cytoplasmic b-catenin as well as evidence of nuclear accumulation. Well-documented WNT pathway proteins were also upregulated, including c-Myc, Connective Tissue Growth Factor (CTGF), Cyclin D1, and WISP2, in mutant HBx-expressing cells, suggesting these mutations act at multiple levels to increase WNT signaling (Figures 4C,D).

Hepatitis B Virus has been shown to increase WNT signaling by upregulating its activating ligand Frizzled7 (FZD7) (Merle et al., 2004). Additional HBV proteins are also thought to participate in this activating pathway. The pre-core protein 22 (p22) is further processed to p17, also known as HBeAg, before being secreted by infected cells (Tran et al., 2020). A human HCC cell line, Huh7, when transfected with p22 upregulated FZD7 to a greater extent than HBx (Tran et al., 2020). Expressing p22 in cell lines without or with known mutations in APC or b-catenin resulted in a synergistic increase in *TCF*/*CTNNB1* transcription that could be reversed with co-expression of DN-TCF4. FZD7 is not the only HBx-induced ligand implicated in activating WNT signaling. Expressing HBx in a normal liver cell line upregulated N1ICD and increased expression of FZD10, but not FZD7 (Sun et al., 2014). Cyclin D1 and b-catenin levels were also upregulated, along with increased cytosolic and nuclear b-catenin. WNT signaling was diminished in cells treated with a Notch inhibitor, or in which *NOTCH1* was knocked down using siRNA approaches. However, delivering *siFZD10* to cells had no effect on NOTCH1 expression, placing NOTCH1 upstream of WNT pathway activation in these cells. Thus, WNT signaling is likely activated by HBx through different mechanisms to promote HCC.

In addition to HBV, HCV can also promote HCC by instilling extended, sequential changes to the liver that occur over time. The HCV genome encodes structural proteins like core, E1, E2, p70 and non-structural proteins namely NS1, NS2, NS3, NS4A, NS4B, NS5A, and NS5B (Mahmoudvand et al., 2019). The HCV core, NS3, NS5A, and NS5B proteins contribute to HCC by interacting with and modulating key host cellular functions, such as cell cycle, proliferation, and apoptosis. When core protein Type 1B or NS4B was expressed in Huh7 cells, or in the normal liver L02 cell line, increased nuclear b-catenin was observed upon activation of WNT3a (Jiang X.H. et al., 2017). This was accompanied by increased expression of *MYC*, *WNT1*, and *CCND1* in Huh7, but not in L02 cells, suggesting these HCV proteins increase WNT signaling to directly affect WNT-mediated cellular processes (Jiang X.H. et al., 2017). The HCV core protein has been shown to inactivate GSK3b, further promoting WNT/b-catenin activity. In the nucleus, b-catenin complexes with TCF to activate downstream targets such as *MYC, CCND1*, and *WISP2*, to enhance cell-cycle progression and cell proliferation (Mahmoudvand et al., 2019). HCV core protein can also act positively to increase WNT/b-catenin signaling by upregulating expression of LRP5/6 and FZD receptors. Increased signaling through these receptors releases b-catenin from E-cadherin complexes, facilitating its translocation to the nucleus, and subsequent transcriptional activity (Figures 4C,D) (Mahmoudvand et al., 2019). To further complicate the predicted effects of core proteins on patient disease progression, Aicher et al. (2018) used HEK293 and Huh7.5 cells to demonstrate core protein sequence-specific effects on the expression of b-catenin and its transcriptional targets, based on the differences in HCV substrains obtained from clinical isolates. Thus, although it is clear that HCV co-opts WNT signaling pathways to promote HCC, it is possible this occurs in a substrain-specific manner, at least with regard to core protein effects.

Hepatitis C virus infection is also associated with promoter hypermethylation of the WNT pathway inhibitors, Secreted Frizzled-related Protein 2 gene (*SFRP2*) and *DKK1*, leading to WNT/b-catenin activation. Studies by Umer et al. (2014) suggest *SFRP2* and *DKK1* hypermethylation may occur early following HCV infection and may lead to HCC *via* a multi-step process. In this study, the authors utilized bisulfite sequencing to analyze liver biopsies from patients with chronic hepatitis, liver cirrhosis, and HCC. Compared to normal liver samples, *SRFP2* and *DKK1* showed significant hypermethylation in HCV-infected patients. Although no differences in *DKK1* methylation was noted between the different conditions, there was a progressive increase in *SFRP2* promoter methylation with normal liver < chronic hepatitis < liver cirrhosis < HCC (Umer et al., 2014). These findings are consistent with those of Eldeeb et al. (2020) which showed that in HCV-infected patients with liver cirrhosis, with or without corresponding HCC, DKK1 expression was significantly decreased. Thus, evaluating promoter methylation status, especially for *SFRP2*, or DKK1 protein levels may prove to be useful for monitoring disease progression following HCV infection (Figure 4C).

In an interesting study, Zhang et al. (2012) examined how HCV infection intersects with miRNA expression. HCV-infected Huh7 cells expressed higher levels of the pro-inflammatory miRNA, miR-155, *via* an NF-kB-mediated pathway. This resulted in greater accumulation of nuclear b-catenin, along with increased expression of cMYC, Cyclin D1, and survivin. Increased miR-155 did not appear to affect GSK3b or AXIN1 signaling. Functionally, high miR-155 expression led to a block in apoptosis and increased cellular proliferatio *in vitro* and increased tumorigenesis *in* nude mice. HCV core protein can also act positively to increase WNT/b-catenin signaling by upregulating expression of LRP5/6 and FZD receptors. Increased signaling through these receptors releases b-catenin from E-cadherin complexes, facilitating its translocation to the nucleus and subsequent transcriptional activity. Taken together, these data point to additional direct and indirect means by which HCV promotes HCC through dysregulated WNT signaling.

### WNT Signaling Is Dysregulated Following Infection With HPV or EBV

HPV codes for multiple viral proteins with critical functions in viral infection, integration and replication. Its capacity to transform human epithelial cells are ascribed to its E6 and E7 proteins, and this includes promoting b-catenin translocation to the nucleus where it acts transcriptionally, to facilitate tumorigenesis. Unlike the actions of HCV and HBV, HPV infection rarely causes mutations in *CTNNB1* or *AXIN1*. However, HPV can activate components of the WNT signaling pathway to facilitate cellular transformation. Microarray gene analysis shows HPV infection induces multiple genes, including upregulation of WNT related proteins (Fragoso-Ontiveros et al., 2012). It has also been shown that in some cervical and oropharyngeal squamous cell cancers, HPV infection reduces the amount of membrane-associated b-catenin while, at the same time, it increases its cytosolic and nuclear accumulation (Rodríguez-Sastre et al., 2005). HPV can further act to increase GSK3b phosphorylation, which can further attenuate its inhibitory action on WNT signaling (Figures 4C,D) (Rath et al., 2015).

Akin to HCV, HPV can also modulate miRNAs expression to affect WNT signaling. Mo et al. (2015) showed HPV can increase expression of miRNAs, the targets of which may repress WNT signaling only during early stages of CC development. The authors suggest that the differential regulation of *AXIN2, DVL3*, and *LEF1* by miR-622, miR-920, and miR-507, respectively, may act to stabilize or increase WNT signaling at later stages of cancer progression (Mo et al., 2015). However, additional functional studies are needed to confirm how differences in miRNA expression contribute to cellular transformation caused by HPV.

Epigenetic changes that modulate the WNT pathway have been observed as a result of HPV infection and, frequently, the WNT inhibitory genes are targeted. For instance, in some HPV associated OC, increased *APC* and *SFRP3* promoter methylation have been observed (Al-Shabanah et al., 2014). In HPV positive CC, hypermethylated *SFRP2* and *DKK3* promoters have also been reported (van der Meide et al., 2011).

Studies suggest HPV can also affect other proteins that crosstalk with the WNT pathway. LRG5 is a G-protein coupled receptor. Chen Q. et al. (2014) demonstrated that, as HPV-induced CC progressed, increased LGR5 expression could be detected *via* immunohistochemistry. Furthermore, *in vitro* reporter assays indicated that LGR5 activates WNT signaling by upregulating c-myc, cyclin D1, and b-catenin to increase cell cycle progression and drive proliferation (Chen Q. et al., 2014). HPV can also modulate less well-known WNT signaling partners like, FOXM1, a novel component of WNT signaling (Chen P.M. et al., 2014). Patients diagnosed with lung or oral cancer showed worse overall and relapse-free survival compared to patients who lacked a demonstrated interaction between b-catenin and FOXM1 (Chen P.M. et al., 2014). Finally, Lichtig et al. (2010) provide evidence that HPV infection may even alter b-catenin proteasomal degradation and this may require E6/E6AP. Thus, HPV infection can successfully promote WNT-mediated epithelial cell transformation through multiple mechanisms to directly or indirectly activate the WNT pathway.

EBV is yet another oncovirus that targets the WNT signaling pathway to transform epithelial cells. Studies using telomerase-immortalized normal oral keratinocytes (NOKs) showed that EBV infection led to epigenetic reprogramming, CpG hypermethylation, and delayed responsiveness to differentiation cues (Birdwell et al., 2014). Using the same system, Birdwell et al. (2018) further investigated the effects of EBV infection on cellular transformation and discovered that the invasive phenotype acquired by NOKs persisted, even after the viral loss. LEF and WNT5a, both of which are elevated in NPC, were increased in EBV-infected NOKs. LEF and WNT5a expression remained high for more than 20 passages, after EBV viral expression was no longer detected. Forced expression of LEF and WNT5a enhanced the invasive capacity of NOKs, while knocking down *LEF1* reduced their invasiveness, even in the presence of WNT5a expression. The data suggest that EBV may provide a selective advantage to infected cells, with LEF1 contributing to their metastatic potential (Birdwell et al., 2018).

The tumor suppressor, *DACT2*, is expressed in various healthy tissues and is a methylation target in some cancers (Zhang et al., 2018). Zhang et al. (2018) demonstrated this WNT regulator was also the target of hypermethylation in EBV-related NPC. Treating NPC cells with the DNA methyltransferase inhibitor, 5-aza-2′-deoxycytidine, restored DACT2 expression to normal levels. Ectopic expression of DACT2 in NPCs also reduced proliferation, migration, and invasion, and induced G2/M arrest by blocking b-catenin/Cdc25 activity (Zhang et al., 2018). Restoring DACT2 expression sensitized NPC cells to the cytotoxic actions of paclitaxel and 5FU, but not to cisplatin, suggesting *DACT2* may be an additional means of modulating the WNT pathway in NPC.

EBV also affects miRNAs to regulate WNT signaling in NPC. The EBV encoded miR-BART22 can induce the cellular miR-4721 through a PI3K/AKT/cMYC/cJUN/Sp1 mediated pathway. GSK3b is a direct target of miR-4721 and in clinical samples, low GSK3b expression correlates with high miR-4721 levels (Tang et al., 2020). Increased miR-4721 further correlates with increased nuclear b-catenin accumulation and greater *CCND1* and *MYC* expression (Tang et al., 2020). Thus, as with other oncogenic viruses, EBV promotes tumorigenesis by subverting WNT signaling through direct and indirect mechanisms.

### Immune Cells as Targets of Oncogenic Viral Transformation

HTLV-1 infection is the underlying cause of ATL in a significant proportion of infected individuals. The virus expresses several proteins known to facilitate leukemic transformation, including the basic leucine zipper (bZIP) factor, HBZ, and the Tax protein; however, they can have opposing effects on WNT signaling. For instance, HBZ and Tax, both can interact with the WNT pathway through a related protein, DAPLE (disheveled-associated protein with a high frequency of leucine residues) (Ma et al., 2013). In the presence of DAPLE, Tax can activate canonical WNT signaling while HBZ suppresses this activation. One way by which HBZ inhibits canonical WNT signaling is by impairing LEF DNA-binding. HBZ can also enhance TGFb-mediated transcription of *WNT5A* to antagonize the canonical WNT pathway (Ma et al., 2013). Knocking down *WNT5A* in HTLV-1-infected cells repressed cellular proliferation and migration, confirming the contribution of WNT signaling to the leukemic process. These contradictory findings suggest that it may be the balance of Tax and HBZ expression following HTLV-1 infection that ultimately tips the scale in favor of leukemogenesis (Figures 4C,D).

To highlight the complex interactions between HTLV-1 and the WNT pathway, it is interesting to note that while Tax functions to promote HTLV-1 viral replication, TCF1 and LEF1 both interact with Tax to attenuate Tax-dependent viral expression and activation of NF-kB and AP-1 (Ma et al., 2015). In contrast, both TCF and LEF are downregulated in activated T cells. Ma et al. (2015) provide *in vivo* data supporting this yin and yang between TCF/LEF expression and viral load using Japanese macaques as a model system. In animals infected with the closely related, Simian T Lymphocyte Virus-1 (STLV-1), a negative correlation between the STLV-1 proviral load and TCF/LEF1 expression was observed in T cells (Ma et al., 2015).

Among its symptoms, patients with severe cases of ATL may exhibit hypercalcemia, bone loss, and bone lesions that are associated with osteoclast-mediated bone resorption (Xiang et al., 2019). One key mediator of these processes is the WNT inhibitor, DKK1, which has been identified as a key regulator of hypercalcemia and bone loss (Colditz et al., 2019). In keeping with the role of Tax protein in modulating WNT signaling, it represses DKK1 in HTLV-1 infected cells (Polakowski et al., 2010). However, DKK1 has also been associated with bone lesions in Multiple Myeloma (Fujita and Janz, 2007). Polakowski et al. (2010) found that HBZ nuclear localization can modulate transcription by binding to p300/CBP transcriptional co-activators. Microarray analysis of cells expressing wild-type or mutant HBZ revealed transcriptional upregulation of *DKK1*, which was attenuated following siRNA knock-down of p300/CBP. Forced HBZ expression in T cells uninfected with HTLV-1 caused *de novo* expression of DKK1, while expressing HBZ in HTLV-1-infected T cells increased its expression (Polakowski et al., 2010). These data are consistent with another study that examined mechanisms of osteolytic bone lesions. Elevated serum levels of DKK1 has also been reported in a mouse model of ATL that expresses HBZ from a granzyme promoter (Esser et al., 2017).

Finally, the γ-herpesvirus KSHV, can target components of the WNT signaling pathway in B cells to promote tumorigenesis (Fujimuro et al., 2003). The LANA protein in KSHV shares homology with AXIN and has been shown to bind GSK3b (Fujimuro and Hayward, 2003). Furthermore, it was shown that interaction with LANA could draw GSK3b away from the inhibitory complex that keeps b-catenin inactive in the cytosol and allows for its nuclear translocation and subsequent transcription of WNT target genes.

Collectively, the data suggest that dysregulating WNT signaling is an effective mechanism by which oncogenic viruses target and manipulate multiple normal cellular processes to facilitate tumorigenesis. Furthermore, these viruses often employ multiple means of circumventing normal WNT signaling. HBV, HCV, EBV, HPV, and KSHV can all inhibit various components of the WNT pathway that normally keep it in its “OFF” state. These same viruses can also enhance positive regulators of WNT signaling, resulting in increased expression of WNT target genes. Thus, by “releasing the brakes and stepping on the gas” of the WNT signaling pathway, oncogenic viruses have evolved to be very efficient at driving cell proliferation, survival, and ultimately, transformation.

## HH, Notch and WNT Pathways: Intersection for Common Therapeutic Targets

**FIGURE 5:**
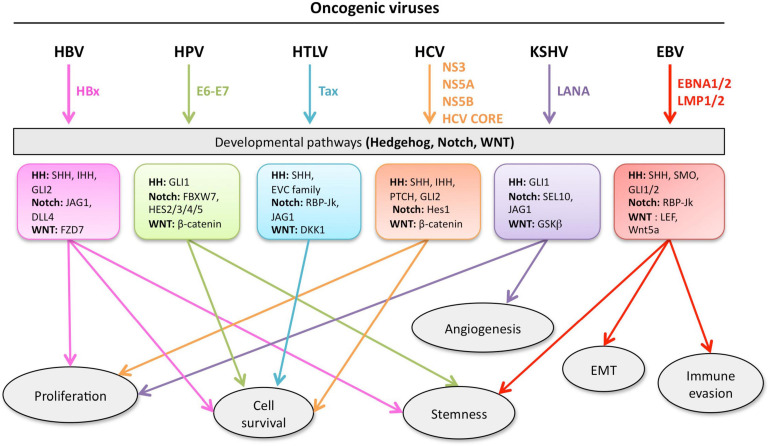
Developmental pathways co-opted during viral oncogenesis. Viruses deregulate developmental signaling to support malignant transformation and disease progression, via a variety of mechanisms, including (but not limited) to, increased proliferation and cell survival, induced stemness to improve fitness of the cancer cells, accrued neo-vascularization, activation of the epithelial-to-mesenchymal transition (EMT), evasion of tumor-targeting immune response. Inhibitors of developmental pathways may prevent the acquisition of new properties by the cancer cells, such as virus-induced transcriptional, metabolic, and functional reprogramming. Each color is associated with a specific virus and the cell processes controlled the developmental pathway.

Given the complexity of the crosstalk between viruses and the multiple members of the developmental pathways, it is of paramount importance to better understand the molecular mechanisms involved in host-pathogen interactions to develop novel therapies. Notwithstanding the multitude of signaling proteins participating in each developmental pathway, the biological outcome is the same, thus leading to proliferation, cell survival, angiogenesis, stemness, EMT, immune evasion, and maintenance of latent viral state. The ability of the same viral protein to connect cellular proteins of the different pathways, such as HPV E6 or E7 with members of the Notch, HH, and WNT pathways raises the question whether simultaneous or ordered interactions occur in enhanced cell proliferation or tumor progression. Conversely, different viral proteins can interact with signaling proteins of the same pathway. For instance, HBx and NS3/5A/5B encoded by HBV and HCV, respectively, interact with the same HH signaling proteins, leading to cell proliferation and survival. GLI1/2 is activated by HBV, HPV, HCV, KSHV, and EBV proteins bringing to cell proliferation and survival, as well as virus immune evasion (Figure 5). Oncogenic JAG1 signaling is induced by HBx, Tax and LANA. The commonly targeted β-catenin by HPV and HCV induces stemness, proliferation and survival in the host cell (Figure 5). Therefore, viral proteins such as HBx, E6-E7, Tax, NS3/5A/5B, LANA, EBNA1/2, and LMP1 could be targeted to block viral communication with the components of each developmental pathway (Figure 5). On the other hand, already available inhibitors of each pathway might be used as therapeutic strategies for virus-associated diseases (Table 1). For instance, GANT-61 inhibited HH pathway members, GLI1 and GLI2 in HPV and KSHV associated tumors (Samarzija and Beard, 2012; Asha et al., 2020). Vismodegib, a SMO inhibitor that effectively terminates HH signaling, decreased liver fibrosis induced by HBV and HCV infection (Kumar et al., 2019). GSI repressed Notch signaling induced by KSHV, EBV, and HCV in associated diseases (Ito et al., 2011; Giunco et al., 2015; Jiang B.C. et al., 2017; Qin et al., 2017). Inhibitors of the WNT pathway decreased oncogenic signaling in EBV and HCV associated diseases (Kimura et al., 2017; Tokunaga et al., 2017; Chan et al., 2019). RIN1 could be used to inhibit RBP-Jk, a Notch pathway molecule exploited by HTLV-1-encoding Tax and EBV-encoding EBNA2, or antibodies against JAG1 might be used to inhibit the oncogenic action of HBV-encoding HBx, HTLV-1-encoding Tax and KSHV-encoding LANA. Similarly, inhibitors against GLI1 and -2 could be used against HBV, HPV, HCV and KSHV associated malignancies and the β-catenin inhibitor (ICG-001), might be used to target HPV and HCV proteins (Table 1 and Figure 5).

**TABLE 1:** Inhibitors of developmental pathways as therapeutic strategies in viral carcinogenesis.

Developmental pathway	Inhibitors	Virus	Disease	Effects	References
Hedgehog	GANT-61	KSHV	Kaposi’s sarcoma	Reduces tumor-sphere formation	Asha et al., 2020
		HPV	Cervical cancer	Decreases proliferation survival and migration	Samarzija and Beard, 2012
	Vismodegib	HBV, HCV	Chronic hepatitis, liver cirrhosis	Decreases liver fibrosis in murine models	Kumar et al., 2019
Notch	GSI X	H1N1	Influenza	Decreases IFNγ production, increases viral load	Ito et al., 2011
	LY-411575	KSHV	Kaposi’s sarcoma	Induces apoptosis of KSHV-infected cells	Curry et al., 2005
	CompE and DBZ	EBV	EBV-associated lymphomas	Induce EBV-lytic cycle, leading to cell death	Giunco et al., 2015
	DAPT	HCV	Chronic hepatitis C	Increases immunosurveillance by down-regulating IL-22, IL-17, Tregs	Jiang B.C. et al., 2017; Qin et al., 2017
Wnt	ICG-001	EBV	NPC	Down-regulates CD44 via β-catenin *in vitro*	Chan et al., 2019
	PRI-724	HCV	HCV-associated fibrosis	Decreases liver fibrosis in humans liver cirrhosis	Kimura et al., 2017; Tokunaga et al., 2017

*NPC, nasopharyngeal carcinoma; EBV, Epstein-Barr Virus; HPV, human papilloma virus; HBV, hepatitis B virus; HCV, hepatitis C virus; KSHV, Kaposi’s Sarcoma associated Herpes Virus; 2,2′-[[Dihydro-2-(4-pyridinyl)-1,3(2H,4H)-pyrimidinediyl]bis(methylene)]bis[*N*,*N*-dimethyl-benzenamine (GANT-61), 2-chloro-N-[4-chloro-3-(2-pyridinyl)phenyl]-4-(methylsulfonyl)benzamide (Vismodegib); γ-secretase inhibitors, GSI X, hydroxyethylene dipeptide isostere LY-411575; Compound E (CompE), dibenzazepine (DBZ) and *N*-[N-(3,5-difluorophenacetyl)-L-alanyl]-(S)-phenylglycine t-butyl ester (DAPT). cAMP-responsive element binding (CREB)-binding protein (CBP) inhibitor (ICG-001) and Potent, specific Wnt pathway inhibitor (PRI-724).*

In addition, developmental pathways may interact with each other to form a complex intertwined network connected by common molecules. Thus, understanding the crosstalk between developmental pathways might help to reveal common, druggable targets. This could be the case with β-catenin, a transcriptional activator of the WNT pathway, which also regulates the Notch-regulated transcriptional repressor, *Hes1* (Borggrefe et al., 2016). Furthermore, there is a direct interaction between Notch1 and β-catenin, the latter having a protective role on Notch1 by reducing its ubiquitination and ultimately activating *Hes1* expression. In addition, during *in vitro* angiogenesis, the protein complex, NICD/RBP-Jk/β-catenin, was formed and directed the differentiation of vascular progenitor cells toward arterial endothelial cells (Yamamizu et al., 2010). In carcinogenesis, WNT signaling activates Notch1 and Notch3 through its ligand, JAG1, in colorectal and OC, respectively (Borggrefe et al., 2016). Crosstalk exists also between HH and Notch pathways. For instance, GLI2 and JAG1 induced expression of each other in OC cells (Steg et al., 2011). Although most studies showed a positive feedback loop between components of the developmental pathways, we cannot exclude cases of negative feedback, especially when designing drugs that target specific common molecules.

Considering the complexity of signaling cascades in each developmental pathway and the existing crosstalk between them, there is a need to carefully design appropriate targeted therapies to avoid adverse, toxic, or off-target drug effects in patients. It will be mandatory to test these drugs in appropriate cellular models *in vitro*, including combined with three-dimensional cell models, which better recapitulate the TME, as well as *in vivo* experiments using robust animal models to fully evaluate how these drugs might influence anti-viral/anti-tumor immune responses in the host.

## Future Perspectives

Developmental pathways are important mediators of the transformation potential of different oncogenic viruses. They can turn normal physiologic pathways into potent carcinogenic routes, either to promote aberrant proliferation and acquisition of stemness, and/or to evade immune surveillance. Future efforts focused on better dissecting the cross-signaling between cellular developmental pathways and viruses will further our understanding of the evolution of viral carcinogenesis, including host-pathogen communications that can also shape the cells in the tumor microenvironment and modulate anti-tumoral immune responses. Given the ability of viruses to behave as forced activators of developmental pathways, we should consider targeting their members in future experimental studies. Viruses can repress signaling pathways at early stages of carcinogenesis, as HPV does with TGFβ and Notch, but can promote later activation, as seen in malignant progression (Meyers et al., 2018). Moreover, as suggested by experimental works in HTLV-1-infected cells and human ATL, inhibitors of developmental pathways may represent drug candidates for intractable human diseases (Takahashi et al., 2014). Taken together, we propose that developmental pathway inhibitors have the potential to attenuate tumor development and further research would underpin their role in combined cancer immunotherapy.

## Author Contributions

SKP and EM researched the literature and performed the literature review and helped in editing the figures. CM, DB, SC, and RP critically revised the manuscript. PT participated to wrote and to edit the manuscript and figures. EA, LM, and MPF put forward the idea of the manuscript, wrote and edited the manuscript, figures, and the table. All authors contributed to the article and approved the submitted version.

## Conflict of Interest

The authors declare that the research was conducted in the absence of any commercial or financial relationships that could be construed as a potential conflict of interest.

## Publisher’s Note

All claims expressed in this article are solely those of the authors and do not necessarily represent those of their affiliated organizations, or those of the publisher, the editors and the reviewers. Any product that may be evaluated in this article, or claim that may be made by its manufacturer, is not guaranteed or endorsed by the publisher.
